# The Jubilee of the Dental Charter

**Published:** 1909-12-11

**Authors:** R. Clement Lucas

**Affiliations:** London, W.


### Editor's Letter-Box.

### THE JUBILEE OF THE DENTAL CHARTER.

Sir,—The dental surgeons have shown themselves desirous of commemorating the jubilee of their charter, and the Royal College of Surgeons of England, as the first examining body to grant degrees in dental surgery, has endeavoured to show its interest in this branch of the profession, first, by accepting the trust of the Odontological Museum, which is now accommodated in a room adjacent to the Hunterian Collection, and secondly, by entertaining the leading members of the profession at a dinner within the precincts of the College. I would venture to suggest that this movement might well be directed towards a more permanent memorial of the jubilee of the dental charter by establishing a research scholarship and demonstratorship in connection with the Dental Museum.

There are already two small endowed prizes, the Tomes and Cartwright prizes, administered by the College, the one awarded every third and the other every fifth year; but the dental profession is deserving of something of a much wider scope than these for the stimulation of research and for the higher teaching of its scientific branches.

My own view favours a far more ambitious scheme than anything in the form of a mere prize, whether awarded after examination or conferred in recognition of original work completed. I would ask for the modest sum of £10,000 to endow a research and teaching demonstratorship in connection with the Dental Museum. In this way not only would a scientific worker be secured, but he would by his demonstrations attract the students from the various dental hospitals to study in the Dental Museum. Such a sum could, I am sure, be easily collected from among the dental surgeons alone; but if they were to impress upon their patients the necessity of endowing dental research there need be no hesitation in raising the sum I have suggested to £50,000, whereby not only research and teaching demonstratorships might be established, but travelling scholarships might be founded which would be of great advantage in keeping English dentistry in touch with that of foreign countries.

It is some twenty-five years ago since I first began to advocate the bringing of the dental surgeons back within the pale of the profession, and giving them the same opportunities as ophthalmic, aural, and other specialists—one could scarcely have anticipated so encouraging a development as has occurred within so short a period. The dental surgeons have not been slow of late to seize the occasions for better recognition; and by placing the Odontological Society under the ægis of the Royal Society of Medicine, and by handing over their superb museum to the care of the Royal College of Surgeons they have shown their desire to be associated with the general body of the medical profession.

Now I think the time has come for the further development of the scientific side of the branch, in which direction several members have already gained great eminence. Should my suggestion find favour with the dental section of our profession, I should be pleased to co-operate with any interested in this project; but it must be distinctly understood that I write quite unofficially, though I have little doubt that the Council of the Royal College of Surgeons would give its consent to the administration of such a research demonstratorship as that proposed, if the money were forthcoming for its endowment.

I am, sir, yours faithfully,

R. Clement Lucas.

